# Efficacy of bio- and neurofeedback for depression: a meta-analysis

**DOI:** 10.1017/S0033291721004396

**Published:** 2022-01

**Authors:** J. Fernández-Alvarez, M. Grassi, D. Colombo, C. Botella, P. Cipresso, G. Perna, G. Riva

**Affiliations:** 1 Department of Psychology, Catholic University of the Sacred Heart, Milan, Italy; 2 Department of Basic Psychology, Clinic and Psychobiology, Universitat Jaume I, Castellón, Spain; 3 Department of Clinical Neurosciences, Hermanas Hospitalarias, Villa San Benedetto Menni Hospital, FoRiPsi, Albese con Cassano, Como, Italy; 4 Department of Biomedical Sciences, Humanitas University, Rozzano, Milan, Italy; 5 Ciber Fisiopatología Obesidad y Nutrición, CB06/03 Instituto Salud Carlos III, Madrid, Spain; 6 Applied Technology for Neuro-Psychology Lab, IRCCS Istituto Auxologico Italiano, Milan, Italy; 7 Department of Psychology, University of Turin, Turin, Italy; 8 Department of Psychiatry and Behavioral Sciences, Miller School of Medicine, University of Miami, Miami, FL, USA; 9 Research Institute of Mental Health and Neuroscience and Department of Psychiatry and Neuropsychology, Faculty of Health, Medicine and Life Sciences, University of Maastricht, Maastricht, the Netherlands

**Keywords:** Depression, biofeedback, neurofeedback, heart rate variability, heart rate variability biofeedback, fMRI neurofeedback, meta-analysis

## Abstract

**Background:**

For many years, biofeedback and neurofeedback have been implemented in the treatment of depression. However, the effectiveness of these techniques on depressive symptomatology is still controversial. Hence, we conducted a meta-analysis of studies extracted from PubMed, Scopus, Web of Science and Embase.

**Methods:**

Two different strings were considered for each of the two objectives of the study: A first group comprising studies patients with major depressive disorder (MDD) and a second group including studies targeting depressive symptomatology reduction in other mental or medical conditions.

**Results:**

In the first group of studies including patients with MDD, the within-group analyses yielded an effect size of Hedges' *g =* 0.717, while the between-group analysis an effect size of Hedges' *g =* 1.050. Moderator analyses indicate that treatment efficacy is only significant when accounting for experimental design, in favor of randomized controlled trials (RCTs) in comparison to non RCTs, whereas the type of neurofeedback, trial design, year of publication, number of sessions, age, sex and quality of study did not influence treatment efficacy. In the second group of studies, a small but significant effect between groups was found (Hedges' *g* = 0.303) in favor of bio- and neurofeedback against control groups. Moderator analyses revealed that treatment efficacy was not moderated by any of the sociodemographic and clinical variables.

**Conclusions:**

Heart rate variability (HRV) biofeedback and neurofeedback are associated with a reduction in self-reported depression. Despite the fact that the field has still a large room for improvement in terms of research quality, the results presented in this study suggests that both modalities may become relevant complementary strategies for the treatment of MDD and depressive symptomatology in the coming years.

Major depressive disorder (MDD) represents a worldwide leading cause of disability, with more than 300 million people affected (WHO, 2017). Not only does it entail a major impact on people's quality of life and social functioning (Angermeyer, Holzinger, Matschinger, & Stengler-Wenzke, 2002; Gili et al., 2013; IsHak et al., 2013; Zuelke et al., 2018), but also MDD is strongly related with a vast array of other mental disorders, mainly anxiety disorders (Watson, 2009), as well as a great number of medical conditions, including chronic physical illnesses (Kang et al., 2015) and neurological diseases (Raskind, 2008).

Although psychotherapy (Cuijpers, Cristea, Karyotaki, Reijnders, & Huibers, 2016), psychopharmacology (Cipriani et al., 2018) and the combination of the previous two (Craighead & Dunlop, 2014) have shown to be efficacious, there is still much room for improvement. Around 40–50% of patients do not respond to treatment (Cuijpers et al., 2014) and a third of those who do respond present relapses (Beshai, Dobson, Bockting, & Quigley, 2011; Burcusa & Iacono, 2007). Besides, it is estimated that 75% of depressed people remain untreated (WHO, 2017). Hence, it is of utmost importance to develop new modalities of treatment that can help to overcome the aforementioned obstacles (Kazdin & Blase, 2011).

In this sense, biofeedback is considered one of the existing mind-body interventions that may foster the bridging of physiological and psychological interventions. Biofeedback techniques entail a signal (e.g. video, audio display or tactile) connected to a physiological process that enables the person to be aware of normally unconscious physiological activity (Browne, 2015). In this sense, individuals are provided with explicit information of a certain psychophysiological process in order to foster its regulation.

Biofeedback has principally been used in the medical realm, although there is also a long-standing tradition of research on biofeedback techniques for mental disorders (Lehrer & Gevirtz, 2014; Sacchet & Gotlib, 2016). In particular, post-traumatic stress disorder and substance use disorder are among the most researched conditions (Schoenberg & David, 2014). Different physiological processes have been implemented for biofeedback procedures, including both the central and autonomous nervous systems. Electromyography biofeedback (EMGB), skin conductance biofeedback or heart rate variability biofeedback (HRVB) are some of the most used peripheral responses, while electroencephalographic (EEG) and functional magnetic resonance imaging neurofeedback (fMRI-NF) are two of the most common techniques using neural activity (Sacchet & Gotlib, 2016).

Ample evidence demonstrated that different psychophysiological processes are impaired in patients with MDD. With regard to the neurocircuitry, functional impairments have been identified in prefrontal, limbic, striatal, thalamic and basal forebrain structures (Price & Drevets, 2010). Of particular importance for neurofeedback, there is consistent evidence from EEG research demonstrating that depressive individuals present higher left-hemispheric alpha activity, including hypoactivation in the left prefrontal area. In this regard, an improvement of the depressive symptomatology has been observed after a neurofeedback-based training of this asymmetry (Linden, 2014). Likewise, neuroimaging and brain structural research indicate that people with depression present several abnormalities. For instance, the amygdala has been identified as an important target in neurofeedback interventions for depression due to its role in emotional processing and responding, interacting with different cortical and subcortical areas and having shown to be a key marker of the onset and recovery of MDD (Young et al., 2018).

Likewise, cardiac activity has proven to greatly contribute to the general physiological dysregulation of depressed patients, and not only to be a correlate of the neural dysregulation (Thayer & Mather, 2018). Heart rate variability (HRV), in particular the high frequency (HF) of the spectral domain, is considered to index cardiac vagal tone and thus to be a relevant marker of MDD. Research in this domain indicates that depression is associated with lower resting HF-HRV and lower LF/HF ratio (Hamilton & Alloy, 2016; Kemp et al., 2010).

Taken together, these results indicate that NF and HRVB constitute two techniques that gather consistent theoretical support to justify a psychophysiological intervention. Indeed, there are a number of qualitative reviews (Hammond, 2005; Linden, 2014; Sacchet & Gotlib, 2016; Young et al., 2018) that have gathered the available studies of neurofeedback and biofeedback for MDD and depressive symptomatology. Nevertheless, to the best of our knowledge, no study has meta-analytically established the extent to which this approach is efficacious for MDD and depressive symptomatology, respectively.

Apart from calculating the overall effect of bio- and neurofeedback interventions for MDD, this study also aims to calculate the effect of all bio- and neurofeedback studies that included depressive symptomatology as a secondary outcome measure in subjects suffering from other conditions than MDD.

## Main research questions

1. What is the pooled evidence for the effectiveness of bio- and neurofeedback for MDD?
2. What is the pooled evidence for the effectiveness of bio- and neurofeedback for depressive symptoms in both medical and mental/psychiatric conditions other than MDD?
3. What moderators explain possible sources of heterogeneity among the effect sizes?

## Materials and methods

The systematic review has been developed in accordance with Preferred Reporting Items for Systematic Reviews and Meta-Analyses (PRISMA) (see Supplementary 1) (Moher et al., 2009).

### Search strategy

First, articles were identified through comprehensive searches of the following databases: PubMed, Scopus, Web of Science and Embase. The last update was in December 2018. References lists of review articles were also considered for potential undetected studies and gray literature has also been examined (for the search string see Appendix 2).

### Eligibility criteria

This study follows a two-step level structure, and thus two different eligibility criteria have been considered. To address the first aim, original articles in English reporting data of the efficacy of bio- and neurofeedback in the treatment of MDD were considered. To select studies, the term “clinical depression” utilized in the DSM 5 (American Psychiatric Association, 2013) was considered, which comprises MDD and dysthymic disorder. All studies that either established a diagnosis of depression using a standardized diagnostic interview (such as the SCID, CIDI, or SCAN) or participants who presented elevated symptoms of depression based on self-report measures were considered for inclusion. )

Studies that included subjects taking psychopharmacology or receiving any other active treatment such as hormone therapy or psychotherapy were excluded.

To address the second aim, studies that measured depressive symptomatology through a psychometrically validated instrument and that presented a condition of bio- or neurofeedback in a randomized controlled trial were considered for inclusion. The objective of including this second layer of studies was to determine the extent to which depressive symptoms are treated through bio- and neurofeedback techniques in other mental disorders and particularly in medical studies. In other words, all studies assessing depressive symptomatology as a secondary outcome measure were comprised.

Unpublished studies, conference papers and proceedings, thesis and articles published in non-peer-reviewed were excluded from the study selection in both searches.

### Study selection procedure

One reviewer completed all database searches for both objectives at the same time. All results were exported to EndNote and duplicates were eliminated. After that, two reviewers (JFA and DC) screened independently all titles and abstracts to identify potentially relevant article for any of the two objectives. Two different folders were created, one for each objective. From the total amount of studies that were included for further examination, the two independent reviewers read full texts to determine if the eligibility criteria were fulfilled. Disagreements were resolved through discussion, and if necessary, a third reviewer was consulted.

### Quality assessment of studies

Cochrane Collaboration Risk of Bias tool has been used to assess sources of bias in randomized controlled trials (RCTs). Our considered criteria entail lack of allocation/concealment, lack of blinding, incomplete accounting of outcome or patient events, and selective outcome reporting.

### Effect size calculation and coding of studies

We estimated the effect size of both the difference in change between the groups as well as the pre-post change within the biofeedback groups by using Hedges' *g*, a variation of Cohen's d which takes into account for biases associated with small sample sizes (Hedges & Olkin, 1985). When the group mean, standard deviation (s.d.), variance or standard error of the mean, and a number of subjects were available for each group, these data were preferably used to calculate the effect size. When some of these data were missing, we looked for other data allowing for the effect size computation, such as unstandardized mean differences, *t* and *p* values. If multiple measurements for depression were used in the same study, a pooled effect size was calculated in order to include in the meta-analyses only a single effect size for each study. The pooled estimate of the effect size was calculated as the average of the different effect sizes of each measure. The variance of the pooled estimate was also calculated as the average of the different variances of the effect sizes; as the correlations among the measures are often not reported in the papers, such approach represents the most conservative way to calculate the pooled effect size variance (i.e. the strategy that leads to the largest variances of the estimate of the pool effect size, assuming a perfect correlation among measures). Similarly, a pooled effect size was calculated and included in the meat-analysis if either multiple biofeedback or control groups were used in the study.

In addition, the following study characteristics were coded and included in the analyses as moderators: (1) sex (% of female subjects in the control group); (2) age (mean age in the control group), (3) length of treatment (number of sessions), (4) type of biofeedback intervention (heart rate variability biofeedback or neurofeedback) (5) year of publication, (6) experimental design (randomized-control trial), (7) methodological quality of studies. Coding of moderators 1–3 may be not possible for all studies. In such case, a “not available” was be inserted.

Two of the authors independently performed the computation of effect sizes and any discrepancy was resolved before analysis, with the involvement of a third author in case of persistent disagreement.

### Meta-analytic statistics

Each study effect size was weighted by its inverse variance (the sum of the within-study variance and an estimate of the between-studies variance), giving a larger weighting to studies with large sample sizes than those with small sample sizes. Before excluding a study because it was not possible to calculate the effect size due to a lack of enough statistical details reported in the paper, we tried to contact the corresponding author (only for studies published less than 10 years ago) asking for the missing details. Otherwise, the study was excluded from the analyses.

The pooled effect sizes were estimated using random-effects models (Restricted Maximum-Likelihood Estimation), with confidence intervals and statistical test calculated with Knapp–Hartung method (Knapp & Hartung, 2003), which hypothesizes potential significant heterogeneity among studies. A significance level of 0.05 was applied.

Q statistic and *I*^2^ index were used to investigate the heterogeneity of the effect sizes among studies. The significance level of the Q statistic was set at 0.1 to adjust for the limited statistical power of this test (Petitti, 2001). *I*^2^ can be interpreted as the percentage of the total variability in a set of effect sizes that cannot be attributed only to the sampling error within studies. If the *Q* statistic resulted significant or *I*^2^ suggested heterogeneity, we checked whether the source of this heterogeneity might have been attributed to one single effect size outlying from all others. In this case, we repeated the meta-analytic analyses one-by-one removing each effect size. The effect size was considered outlying when its exclusion from the analyses yielded a resolution of the heterogeneity, and its inclusion during the removal of other effect sizes did not.

Finally, moderators were included in the meta-analysis in order to try explaining possible sources of heterogeneity among the effect sizes. Moderators were considered only if they were available in at least four independent studies. Considering the limited number of studies expected to be included in the current meta-analyses, moderator analysis will be performed separately for each moderator.

### Publication bias

Publication bias was assessed by visual inspection of the funnel plots and by the Egger's regression test (one-tailed *p* of <0.05 was considered to indicate the presence of the bias) (Egger, Davey Smith, Schneider, & Minder, 1997). We also used the trim-and-fill method from Duval and Tweedie (Peters, Sutton, Jones, Abrams, & Rushton, 2007) to determine the nature of potential publication bias and to compute an estimated effect size that accounts for it.

## Results

### Included studies

**Fig. 1.**
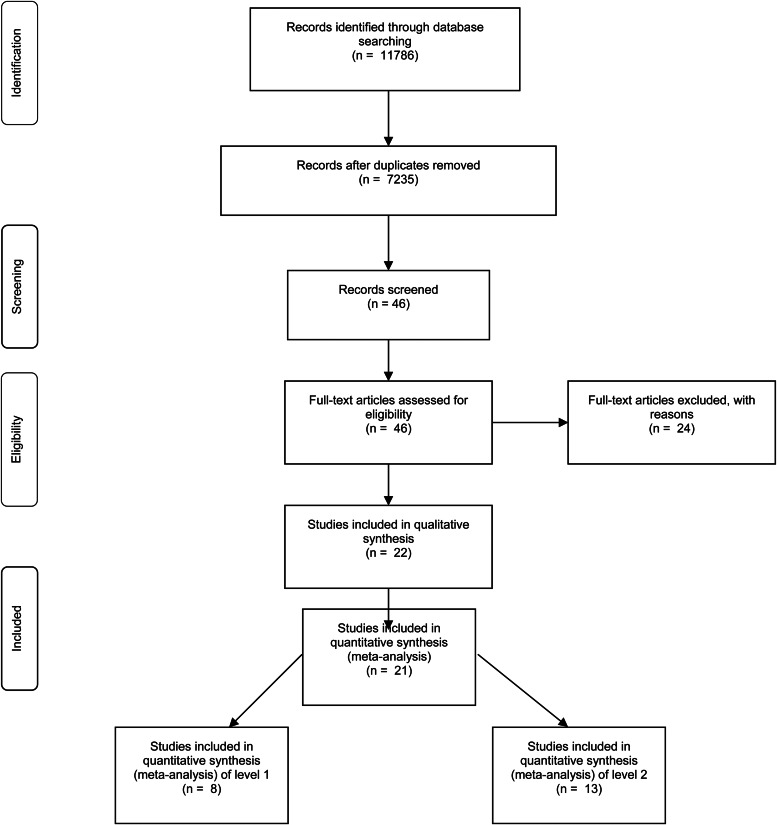
Flowchart of included studies in the meta-analysis.

As illustrated in the PRISMA flow chart (Fig. 1), a total of 11 786 records have been retrieved from the initial database searches. After removing all duplicated articles, the first screening step (examination of titles and abstracts) identified 7235 references that were of potential interest for our meta-analysis. Such a process was carried out by two reviewers and yielded 18 and 24 references, for each of the respective steps of our study. A total number of 22 papers fulfilled the inclusion criteria and were finally included in the study. 24 papers were excluded because they did not satisfy the inclusion criteria (described in Fig. 1). The whole procedure was independently done by two reviewers (JFA and DC).

A flowchart of the general inclusion procedure is reported in Fig. 1. No reply was received from any author we contacted to obtain missing data. Descriptions of all the included studies with relevant variables and study-level characteristics coded for each study are reported in Tables 1–3, for each step of the study.

**Table 1.** Between effect sizes of Neuro- and biofeedback for depressive symptomatology in all conditions

	Country	Type of BF	Control group	Design	Mean age BF	Mean age CG	Sample size (BF/CG)	% of female BF	% of female CG	Instrument	Nr. of sessions	Risk of bias (A/C; LB; IA; SOR)
Young et al. (2017)	USA	fMRI	Placebo	RCT/Double-Blind, placebo-controlled	32	31	36 (19/17)	66%	75%	Composite of 3 scales (HDRS; MADRS; BDI-II)	2	+ / + / + / +
Choi et al. (2011)	South Korea	EEG	Placebo	RCT/Single-blind	28.46	28.54	23 (12/11)	20%	57%	Composite of 2 scales (HDRS & BDI-II)	5	+ /U/ + / +
Li et al. (2015)	China/USA	fMRI	Regular rehabilitation^a^	RCT	54.38	59.64	24 (13/11)	62%	27%	HDRS	3 times/w. for 4–6 w	U/U/U/ +
Caldwell and Steffen (2018)	USA	HRV	Psychotherapy active	RCT	20.09	20.64	20 (10/10)	100%	100%	Beck Depression Inventory-II	5	U/U/U/ +
Yuan et al. (2014)	USA	fMRI	NF based on Left HIPS	Case–control	38	35	27 (13/14)	79%	85%	HDRS	1	+ /U/ + / +
Linden et al. (2012)	UK/Netherlands	fMIR & EEG	Healthy subjects / imagery procedure	Experimental design	48.37	48.5	16 (8/8)	0%	37%	HDRS-17	4	U/U/U/ +
Zotev et al. (2016)	USA	fMRI	Placebo	RANDOMIZED/Double-Blind, placebo-controlled	41	34	13	69%	85%	Profile of Mood States	2	+ /U/ + / +
Mehler et al. (2018)	Netherlands	fMRI	Activation of higher visual processes	.RCT	47.19	46.94	32 (16/16)	69%	62,5%	HDRS-17	5	+ / + / + / +

A/C, Allocation/concealment; Beck Depression Inventory–II; BF, biofeedback; CG, Control group; EEG, electroencephalogram; fMRI, functional magnetic resonance imaging; HAD, Hamilton Depression Rating Scale; HIPS, horizontal segment of intraparietal sulcus; HRV, Heart rate variability; IA, Incomplete accounting of outcome or patient events; LB, Lack of blinding; MADRS, Montgomery-Åsberg Depression; RCT, randomized controlled trial; Nr. of sessions, Number of sessions; SOR, Selective Outcome Reporting.

a Hamilton Depression Rating Scale (17-items).

**Table 2.** Within effect sizes of Neuro- and biofeedback for depressive symptomatology in all conditions

	Country	Type of BF	Design	Mean age	Sample size	Percent female	Instrument	Nr. of sessions	Risk of bias (A/C; LB; IA; SOR)
Young et al. (2017)	USA	fMRI	RANDOMIZED/Double-Blind, placebo-controlled	32	19	66%/75%	Composite of 3 scales (HDRS; MADRS; BDI-II)	2	+ / + / + / +
Choi et al. (2011)	South Korea	EEG	RANDOMIZED/Single-blind	28.46	12	20%/57%	Composite of 2 scales (HDRS & BDI-II)	5	+ /U/ + / +
Yuan et al. (2014)	USA	fMRI	Case–control	38	13	79%/85%	Hamilton Depression Rating Scale	1	+ /U/ + / +
Zotev et al. (2016)	USA	fMRI	RANDOMIZED/Double-Blind, placebo-controlled	41	13	69%/85%	Profile of Mood States	2	+ /U/ + / +

A/C, Allocation/concealment; BF, biofeedback; CG, Control group; EEG, electroencephalogram; fMRI, functional magnetic resonance imaging; IA, Incomplete accounting of outcome or patient events; LB, Lack of blinding; RCT, randomized controlled trial; Nr. of sessions, Number of sessions; SOR, Selective Outcome Reporting.

**Table 3.** Between effect sizes of Neuro- and biofeedback for depressive symptomatology in all conditions

	Country	Type of BF	Comparison condition	Population	Sample size	Percent female	Instrument	Nr. of sessions	Risk of bias (A/C; LB; IA; SOR)
Hallman et al. (2011)	Sweden	HRV	Control (check if WL)	Chronic neck pain	23 (HRV 12; CG 11)	91%	HADS	10	U/U/ + / +
Patron et al. (2013)	Italy	HRV	Daily counseling sessions	After cardiac surgery patients	26 (HRV 13; CG 13)	22.5%	CES-D	5 (45’)	U/U/U/ +
Penzlin et al. (2015)	Germany/USA	HRV	CBT	Addiction (alcohol)	43 (HRV 24; CG 19)	¿?¿?	BDI-II	6 (20’)	+ / + / + / +
Ratanasiripong et al. (2015)	USA/Thailand	HRV	WL	Healthy population	60 (HRV 30; CG 30)	97%	CES-D	1^a^	+ /U/U/ +
Swanson et al. (2009)	USA	HRV	Quasi-false alpha-theta biofeedback	Heart Failure	29 (HRV 15; CG 14)	80%	CES-D	6 (45’)	+ /U/ + / +
Zucker et al. (2009)	USA	HRV	Progressive muscle relaxation	PTSD	38 (HRV 19; CG 19)	44.7%	BDI-II	4 weeks once a day for 20’ at home	+ / + /U/ +
Schönberg et al. (2017)	Germany	Neuro	Sham neurofeedback	ADHD	75 (NBF 37; CG 38)	44%	BDI-II		+ / + / + / +
Schönberg et al. (2017)	Germany	Neuro	Meta-cognitive group therapy group	ADHD	75 (NBF 37; CG 38)	44%	BDI-II	30	+ / + / + / +
Hsueh et al. (2016)	Taiwan	Neuro	Sham neurofeedback	Memory enhancem.	25; 25	60%	BDI-II	12	+ / + /U/ +
Lackner et al. (2016)	Austria / UK	Neuro	Training sessions (different psychotherapeutic aspects)	Addiction (alcohol)	25 (NF 13; CG 12)	0%	BDI-V (modified version of BDI)	12	+ / + /U/ +
Menella et al. (2017)	Italy	Neuro	Active control training	Healthy population	32 (NF 16; CG 16)	100%	BDI-II	7 (45’)	+ / + /U/ +
Dehghani-Arani et al. (2013)	Iran	Neuro	TAU (medication)	Addiction (opiate)	20 (NF 10; CG 10)	No info	GHQ-28	30	+ / + /U/ +
Choobforoushzadeh et al. (2015)	Iran	Neuro	TAU (medication)	Multiple schlerosis	24 (NF 12; CG 12)	50%	HADS	16	+ / + /U/ +
Yu et al. (2018)	Taiwan	HRV	Medicine	Coronary Artery Disease	210 (105 HRV; 105 CG)	12% HRV 10.20%CG	BDI-II	6 (60’)	+ /U/ + / +

A/C, Allocation/concealment; ADHD, Attention Deficit Hyperactivity Disorder; BDI, Beck Depression Inventory; CES-D, Center for Epidemiologic Studies Depression Scale; CG, Control group; GHQ, General Health Questionnaire; HADS, Hospital Anxiety and Depression Scale; HRV, heart rate variability; IA, Incomplete accounting of outcome or patient events; PTSD, Post Traumatic Stress Disorder; RCT, randomized controlled trial; NF, Neurofeedback; Nr. of sessions, Number of sessions; LB, Lack of blinding; SOR, Selective Outcome Reporting TAU, Treatment as usual; Waiting List.

a One session with therapists and then four weeks to use at home three times a day.

### Efficacy of bio- and neurofeedback for MDD (level 1)

#### Pre-post between-group effect sizes

**Fig. 2.**
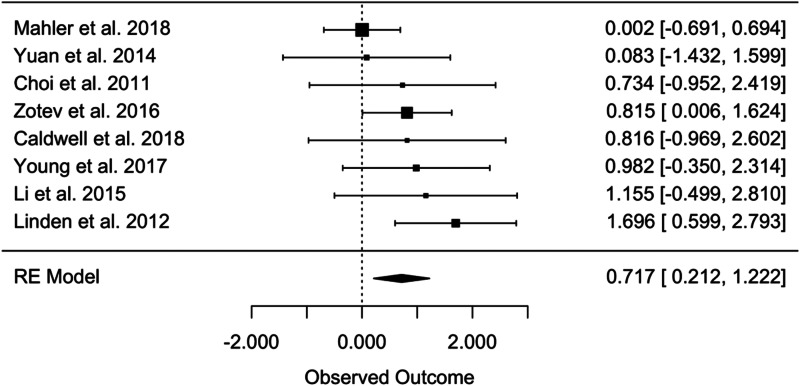
Pre-post between-group effect sizes in level 1.

For the pre-post between-group analysis comparing the bio- and neurofeedback and control groups, the random-effects analyses yielded an overall effect size of Hedges' *g* = 0.717 (95% CI 0.2121–1.1224, *t* = 3.357, *p* = 0.0121) (Fig. 2), indicating a greater efficacy of bio- and neurofeedback compared to control treatments in the treatment of MDD. In total, this meta-analysis was based on eight studies and 176 patients. No evidence of significant heterogeneity was found considering the Q statistics (*Q* = 8.193, *p* = 0.316), while *I*^2^ resulted of 29%, indicating a small potential heterogeneity among the effect sizes of the single studies (Higgins, 2003).

#### Moderator analyses and publication bias

**Fig. 3.**
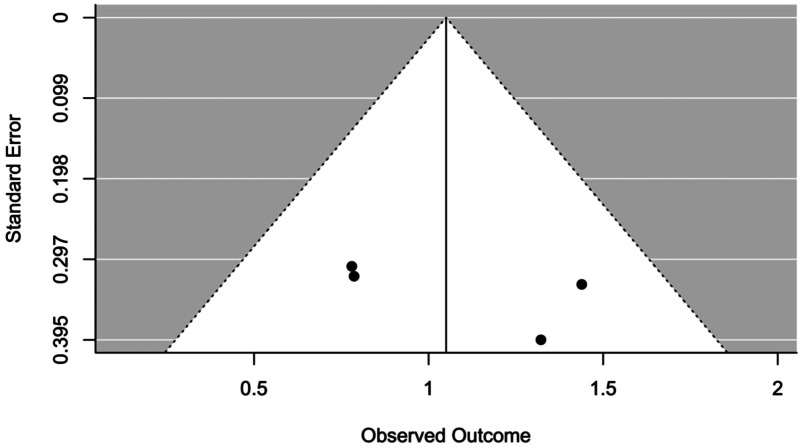
Funnel plot between analyses in level 1.

The effect of all moderators resulted statistically non-significant (see Table 4). The occurrence of publication bias was not suggested by any of the tests used (Trim and Fill analysis) suggests that no studies needed to fall to the right or left of the mean to make the plot symmetrical, and Egger's test resulted not significant (*p* = 0.4365) as well as by visual inspection of the funnel plot (Fig. 3).

#### Pre-post within-group effect sizes

**Fig. 4.**
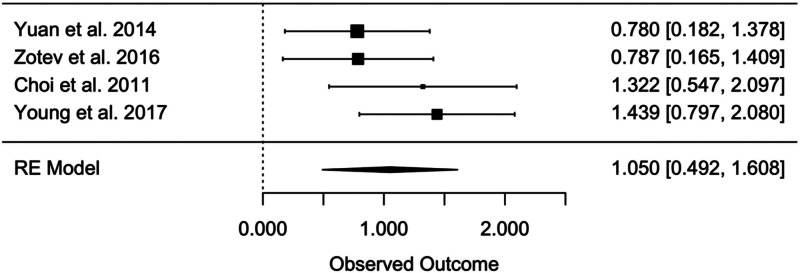
Pre-post within-group effect sizes in level 1.

For the pre-post within-group analysis of the sole biofeedback treatment, the random effects meta-analysis yielded an overall within-group effect size of Hedges' *g* = 1.050 (95% CI 0.492–1.608, *t* = 5.991, *p* = 0.001) (Fig. 4), which indicates a significant efficacy of bio- and neurofeedback in improving MDD symptomatology (*n* = 110). No evidence of significant heterogeneity was found considering the *Q* statistics (*Q* = 3.353, *p* = 0.340), and also *I*^2^ (13%) suggests a very limited heterogeneity among the effect sizes of the single studies.

#### Moderator analyses

Among the moderators (see Table 5), only the experimental design resulted in having a significant effect in moderating the overall within-group effect size (*F* = 126.582, *p* = 0.008). The within-group effect size of biofeedback treatments resulted significant both in randomized-controlled studies (Hedges' g = 1.391, 95% CI 1.216–1.566, *t* = 34.164, *p* < 0.001) and in non-randomized studies (Hedges' *g* = 0.783, 95% CI 0.630–1.566, *t* = 22.045, *p* = 0.002), with the latter resulting significantly greater than the former.

**Table 4.** Moderators between analysis level 1

	INTERCEPT							MODERATOR						
MODERATOR	Interpretation of the estimate	Estimate	se	*t*	*p*	95% CI lower bound	95% CI higher bound	Interpretation of the estimate	Estimate	se	tval	pval	95% CI lower bound	95% CI higher bound
Type of feedback	Estimated effect size of hrv biofeedback	0.996	0.591	1.686	0.143	−0.450	2.443	Estimated effect size difference between neurofeedback and hrv biofeedback	−0.321	0.640	−0.503	0.633	−1.886	1.243
Randomized control trial	Estimated effect size of non-**randomized** control trials	0.958	0.322	2.973	0.025	0.170	1.747	Estimated effect size difference between randomized and non-randomized control trials	−0.452	0.430	−1.051	0.334	−1.503	0.600
Year of publication	Estimated effect size for studies published in 2011	1.459	0.406	3.593	0.011	0.465	2.452	Estimated change of the effect size for every year of publication after 2011	−0.167	0.076	−2.214	0.069	−0.352	0.018
Number of sessions	Estimated effect size with 4 sessions	0.717	0.228	3.144	0.020	0.159	1.276	Estimated change effect size change for every additional session	0.025	0.066	0.379	0.718	−0.136	0.186
Average age of biofeedback group	Estimated effect size at average age of 40 years	0.724	0.230	3.144	0.020	0.161	1.288	Estimated effect size change for every additional year of the mean age	0.003	0.025	0.109	0.917	−0.059	0.064
Pecentage of female subjects in the biofeedback group	Estimated effect size with only male subjects	1.473	0.419	3.518	0.013	0.448	2.498	Estimated effect size difference between only female and only male subjects	−1.362	0.651	−2.092	0.081	−2.954	0.231
Quality of the study	Estimated effect size with low risk of bias	0.210	0.284	0.741	0.487	−0.485	0.906	Estimated effect size difference effect size between unknown/high and low risk of bias	0.738	0.369	1.997	0.093	−0.166	1.642

**Table 5.** Moderators within analysis level 1

MODERATOR	INTERCEPT							MODERATOR						
	Interpretation of the estimate	E	se	*t*	*p*	95% CI lower bound	95% CI higher bound	Interpretation of the estimate	estimate	se	tval	pval	95% CI lower bound	95% CI higher bound
Type of neurofeedback	*all studies investigated neurofeedback efficacy*													
Randomized control trial	Estimated effect size of non-randomized control trials	0.78	0.034	22 045	0.002	0.630	0.936	Estimated effect size difference between randomized and non-randomized control trials	0.608	0.054	11 251	0.008	0.375	0.840
Year of publication	Estimated effect size for studies published in 2011	1.06	0.425	2.511	0.129	−0.761	2.896	Estimated change of the effect size for every year of publication after 2011	−0.002	0.099	−0.023	0.984	−0.429	0.424
Number of sessions	Estimated effect size with 4 sessions	1.26	0.290	4.343	0.049	0.012	2.511	Estimated change effect size change for every additional session	0.122	0.131	0.926	0.452	−0.443	0.686
Average age of biofeedback group	Estimated effect size at average age of 40 years	0.79	0.129	6.142	0.025	0.236	1.343	Estimated effect size change for every additional year of the mean age	−0.058	0.020	−2.914	0.100	−0.144	0.028
Percentage of female subjects in the biofeedback group	Estimated effect size with only male subjects	1.56	0.566	2.766	0.110	−0.870	4.004	Estimated effect size difference between only female and only male subjects	−0.832	0.864	−0.963	0.437	−4.551	2.887
Quality of the study	Estimated effect size with low risk of bias	1.44	0.276	5.221	0.035	0.253	2.624	Estimated effect size difference effect size between unknown/high and low risk of bias	−0.528	0.320	−1.653	0.240	−1.903	0.847

**Table 6.** Moderators level 2

MODERATOR	INTERCEPT							MODERATOR						
	Interpretation of the estimate	Estimate	se	*t*	*p*	95% CI lower bound	95% CI higher bound	Interpretation of the estimate	Estimate	se	tval	pval	95% CI lower bound	95% CI higher bound
Type of neurofeedback	Estimated effect size of hrv biofeedback	0.28	0.112	2.506	0.026	0.039	0.522	Estimated effect size difference between neurofeedback and hrv biofeedback	0.057	0.180	0.320	0.754	−0.331	0.446
Randomized controlled trial	*all studies are RCTs*													
Year of publication	Estimated effect size for studies published in 2011	0.24	0.173	1.413	0.181	−0.129	0.619	Estimated change of the effect size for every year of publication after 2011	0.012	0.032	0.387	0.705	−0.056	0.081
Number of sessions	Estimated effect size with 4 sessions	0.26	0.122	2.144	0.055	−0.007	0.532	Estimated change effect size change for every additional session	0.000	0.011	0.015	0.988	−0.024	0.025
Average age of biofeedback group	Estimated effect size at average age of 40 years	0.29	0.108	2.683	0.023	0.049	0.530	Estimated effect size change for every additional year of the mean age	0.000	0.007	−0.017	0.986	−0.015	0.015
Percentage of female subjects in the biofeedback group	Estimated effect size with only male subjects	0.20	0.122	1.657	0.128	−0.070	0.475	Estimated effect size difference between only female and only male subjects	0.013	0.017	0.725	0.485	−0.026	0.052
Quality of the study	Estimated effect size with low risk of bias	0.01	0.198	0.036	0.972	−0.424	0.438	Estimated effect size difference effect size between unknown/high and low risk of bias	0.351	0.218	1.609	0.134	−0.124	0.827

#### Publication bias

**Fig. 5.**
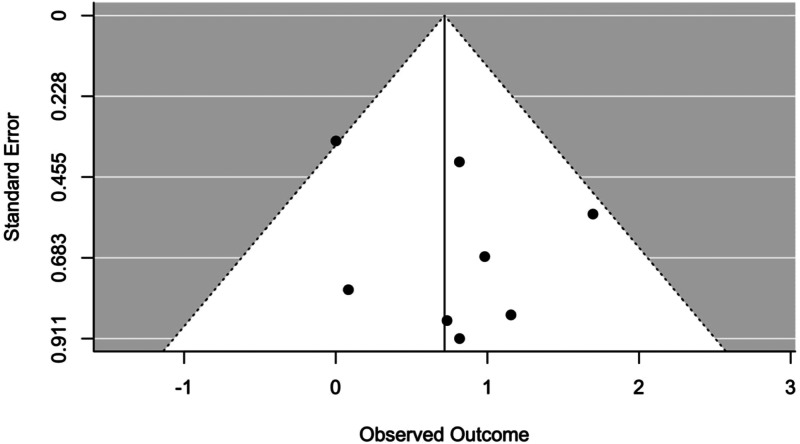
Funnel plot within analyses in level 1.

Trim and Fill analysis indicated that one study would need to fall to the left of the mean to make the plot symmetrical, while no studies on the other side. This suggests that the overall effect size calculated in the within-group analysis may be inflated by the lack of inclusion in the meta-analysis of some unreported study, as it is also evidenced by visual inspection of the funnel plot (Fig. 5). However, the random-effects meta-analysis performed adjusting for missing studies still yielded a significant overall effect size (Hedges' *g* = 1.196, 95% CI 0.985–1.407, *t* = 11.102, *p* < 0.0001), with only a very small reduction of its previous magnitude. Instead, no evidence of publication bias was suggested by the Egger's test (*p* = 0.281)

### Efficacy of biofeedback for depressive symptoms in other conditions (level 2)

#### Pre-post between-group effect sizes

**Fig. 6.**
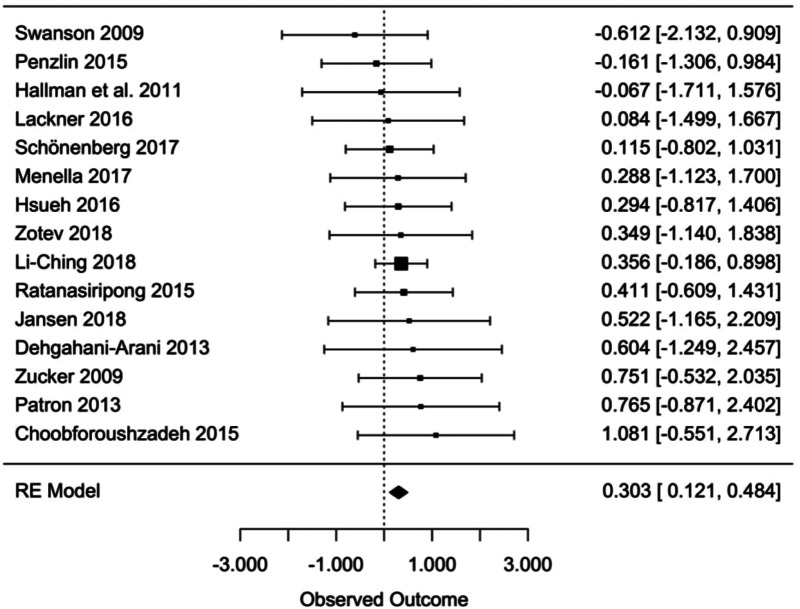
Pre-post between-group effect sizes in level 2.

For the pre-post between-groups analysis comparing the bio- and neurofeedback and control groups, the random-effects analyses yielded a significant overall effect size of Hedges' *g* = 0.303 (95% CI 0.121–0.484, *t* = 2.217, *p* = 0.003) (Fig. 6), indicating a greater efficacy of bio- and neurofeedback compared to control treatments for depressive symptoms (*n* = 736). No evidence of significant heterogeneity was found considering the *Q* statistics (*Q* = 4.350, *p* = 0.993), and also *I*^2^ (0%) suggests a lack of heterogeneity among the effect sizes of the single studies.

#### Moderator analyses and publication bias

**Fig. 7.**
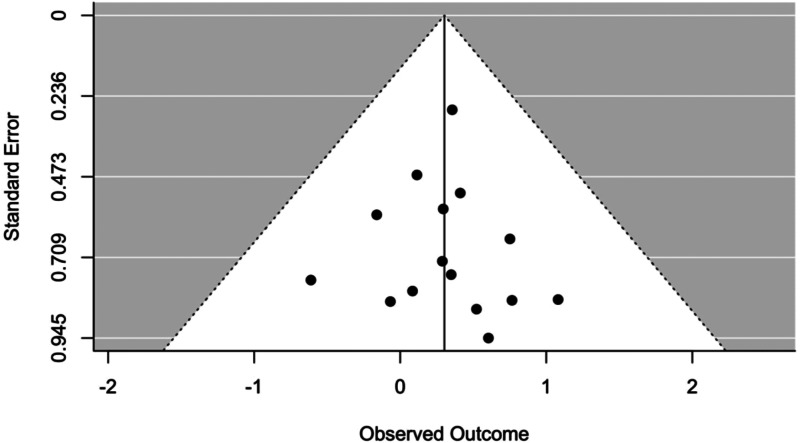
Funnel plot between analyses in level 2.

The effect of all moderators resulted statistically non-significant (Table 6) and no occurrence of publication bias was suggested by any of the tests used (Trim and Fill analysis suggests that no studies needed to fall to the right or left of the mean to make the plot symmetrical, and Egger's test resulted not significant with a *p* = 0.911) as well as by visual inspection of the funnel plot (Fig. 7).

#### Pre-post within-group effect sizes

Pre-post within-group analysis of the sole biofeedback treatments could not be performed because only one study (Kayiran, Dursun, Dursun, Ermutlu, & Karamürsel, 2010) satisfied all the inclusion criteria for this part of the meta-analysis.

## Discussion

To our knowledge, this is the first meta-analysis of bio- and neurofeedback techniques for the treatment of MDD and depressive symptoms. Taken together, these findings suggest that bio- and neurofeedback constitute effective interventions for both individuals with clinical depression and with secondary depressive symptomatology. Besides, the results are in line with the findings of previous qualitative reviews (Hammond, 2005; Linden, 2014; Sacchet & Gotlib, 2016; Young et al., 2018).

### Bio- and neurofeedback for MDD

While the within-group analyses yielded an effect size of Hedges' *g* *=* 0.717, the between-group analysis revealed an effect size of Hedges' *g* *=* 1.050. The moderator analyses indicate that treatment efficacy is only significant when accounting for experimental design, in favor of RCTs in comparison to non-RCTs. The fact that all other moderators were non-significant may indicate that results can be generalized to a range of social and clinical characteristics.

Nevertheless, these results must be taken with caution given that only one RCT has been conducted for MDD using HRVB (Caldwell & Steffen, 2018), and thus making it difficult to conclude that this technique is effective for MDD. Additionally, many of the identified studies targeting clinical depression with HRVB could not be included due to several reasons, such as the absence of a control group or the fact that participants were taking antidepressants. So far, a considerable number of studies have previously indicated that HRVB is effective for depression, frequently referring to the research conducted by Karavidas et al. (2007) or Siepmann, Aykac, Unterdörfer, Petrowski, and Mueck-Weymann (2008). While it is true that both interventions were effective, none of them had either a control condition, in both cases patients were under psychopharmacological treatment and the samples were underpowered.

Conversely, neurofeedback gathered more evidence. The existing studies for neurofeedback comprised both studies brought forth for EEG (Cheon, Koo, & Choi, 2016; Choi et al., 2011) and fMRI (Li et al., 2015; Linden et al., 2012; Mehler et al., 2018; Young et al., 2014, 2017). Among the EEG studies, the target was to regulate the frontal asymmetric activation increasing the relative left frontal activity and consequently to improve depressive symptomatology. In the case of the fMRI studies, some of them targeted the amygdala (Young et al., 2014, 2017) and others the insula and lateral prefrontal areas (Mahler et al., 2018), all of which are involved in the regulation of emotions (Sebastian & Ahmed 2018). From this point of view, emotion regulation is a well-established transdiagnostic factor that explains both the appearance and maintenance of a vast array of affective disorders (Aldao, Nolen-Hoeksema, & Schweizer, 2010), including MDD and depressive symptoms. Integrating the evidence from the prominent theoretical frameworks, it is, therefore, consistent to expect that regulating the principal physiological substrates associated with emotion regulation might increase the functionality of brain regions involved in several affective disorders, and thus decreasing the associated symptomatology.

Apart from RCTs, there are numerous single cases studies available in the literature, all conducted with EEG biofeedback (Baehr, Rosenfeld & Baehr, 1998, 2001; Earnest, 1999; Grin-Yastenko et al., 2018; Hammond, 2000; Rosenfeld, 2000). Besides, there are a set of studies that have focused on cognitive, affective or physiological variables without measuring depressive symptomatology but are worth mentioning given that they show the improvement of MDD with neurofeedback. Illustrative examples are the works by Escolano et al. (2014) for the regulation of cognitive deficits through the regulation of alpha activity or the study by Hamilton and Alloy (2016), in which the authors demonstrated the efficacy in reducing the activity in the salience network. Last but not least, the cutting-edge research carried out by the Laureate Institute for Brain Research, who studied the role of fMRI for the increase of amygdala functional connectivity (Young et al., 2018) or correlation between amygdala activity and EEG asymmetry during emotion regulation (Zotev et al., 2016).

Taken together, the results of neurofeedback for MDD indicate that it constitutes a promissory therapeutic alternative. Nonetheless, the discussion around neurofeedback is currently a matter of controversy in the scientific community. Specifically, a fierce discussion has arisen with regard to the consistency of results derived from EEG neurofeedback (EEG-nf). While some authors claim that EEG-nf has a too broad therapeutic target and thus it is not possible to disentangle specific from placebo effects (Thibault, Lifshitz & Raz, 2016; Thibault & Raz, 2017, 2018), others defend a more nuanced position (Micolaud Franchi & Fovet, 2018). In any case, for the specific case of depression, it is clear that more studies are required before establishing a concluding statement given that the existing evidence is scant.

According to the results of the meta-analysis, MDD has been successfully treated either with HRVB or neurofeedback. Specifically, all studies comprised in level 1 achieved positive results in their patients. This is consistent with the extant theories explaining the mechanisms behind the psychophysiological dysfunction in affective disorders. Several theoretical frameworks such as the Polyvagal theory (Porges, 2007), the Neurovisceral integration model (Thayer & Lane, 2009) or the baroreflex theory (Lehrer & Gevirtz, 2014) explored the relationship between visceral signals, afferent systems, and brain activity. Albeit there are differences among these models, there is a common understanding regarding a reciprocal determination between parasympathetic activity (in particular HF-HRV) and brain activity (in particular cortical regions like the prefrontal cortex and subcortical regions like the amygdala). In line with this, there is increasing evidence supporting that HRV and certain breathing patterns might have a causal role on the regulatory brain networks involved in emotion regulation (e.g. Thayer & Mather, 2018).

### Bio- and neurofeedback for depressive symptomatology

Regarding this second group of studies, the effect size was small in magnitude, albeit significant, and smaller than the pre-post between-groups effect size found for MDD. However, it can be stated for the first time that bio- and neurofeedback techniques are efficacious for the reduction of depressive symptomatology. Given the fact that many of the included studies presented heterogeneous conditions, different baseline levels of depressive symptoms and types of biofeedback, the conclusions should be taken with caution. Moreover, even if many of the included studies presented heterogeneous conditions, no evidence of significant heterogeneity among the effect sizes of the different studies was found. This suggests a quite stable efficacy of bio- and neurofeedback on depressive symptoms independently from the condition.

### Gaps and future challenges in the literature of bio- and neurofeedback for depression

First, it must be clearly stated that only a few of the included studies were rigorously conducted in both groups. The risk of bias was high or unclear in the majority of the studies, which represents an undoubted necessity of enhancing the quality of research in this field.

Besides, regarding the design, some flaws were identified. First, a great part of the studies was underpowered. Given the increasing availability of low cost but reliable psychophysiological devices, bigger samples will be possible to be recruited in the near future. This would represent an important step in order to more clearly determine the extent to which bio- and neurofeedback are effective interventions. In this direction, our results suggest the need for rigorous RCTs. Due to the high costs of conducting RCTs, single-case experimental designs appear also as a good alternative (Bentley, Kleiman, Elliott, Huffman, & Nock, 2019; Kazdin, 2019). Furthermore, future studies should also consider the inclusion of follow-up assessments. Given that depression usually has a high risk of recurrence (Burcusa & Iacono, 2007), the stability of the therapeutic gains in the mid and long term is of paramount importance.

A third important aspect regarding the design of the studies revolves around control groups. Only a few studies presented active conditions as comparators and even fewer studies included both an active and a wait-list condition. Bio- and neurofeedback techniques permit to easily implement sham conditions. This may allow to increase the experimental rigor and thus to more accurately determine the specific contribution of the active aspects in the final outcome.

A fourth aspect to mention in the primary studies, also identified by Goessl, Curtiss, and Hofmann (2017), is the necessity to better specify the amount of time spent with the professional or practicing bio- or neurofeedback and the therapeutic protocols that were used. The dose-response relationship may provide clues to explain mechanisms of change, something that has been scarcely researched in bio- and neurofeedback yet.

In the present meta-analysis, all included studies were carried out with traditional bio- and neurofeedback methods. That is, presenting the physiological process in a visual manner but without transforming the sensing into a particular actuation output that may be more relevant or engaging for the participants (Kitson, Prpa & Riecke, 2018). Besides, the field of bio- and neurofeedback has a lot of potentialities if different technologies are integrated into classical procedures. New engineering and design developments may foster multimodal biofeedback systems, taking into account auditory, visual and haptic feedbacks (Bergstrom, Seinfeld, Arroyo-Palacios, Slater, & Sanchez-Vives, 2014; Jones & Sarter, 2008). From this point of view and with regard to depression, a recent study has explored how music neurofeedback (EEG) could improve symptomatology in elderly people (Ramirez, Palencia-Lefler, Giraldo, & Vamvakousis, 2015). Also, the cross-integration of biofeedback, virtual reality, and serious games is emerging (Schoeller et al., 2019). Some ongoing examples for affective regulation are already available, such as gamified biofeedback in mobile devices for stress management (Dillon, Kelly, Robertson, & Robertson, 2016) or virtual reality-based biofeedback for generalized anxiety disorder (Repetto et al., 2013). Novel advancements are also very relevant, as the combination of biofeedback in a mobile-based application for the synchronization of HRV and electroencephalography (Lin, 2018).

Finally, machine-learning techniques (MLT) may help to personalize bio- and neurofeedback. Adapted features, feedback, and mental strategies could allow for more tailored interventions based on the characteristics of the user (Alkoby, Abu-Rmileh, Shriki, & Todder, 2018; Perna, Grassi, Caldirola, & Nemeroff, 2018). In the case of neurofeedback, the complexity of neural patterns suggests the convenience of adopting statistical strategies that can foster the identification of individual patterns. Specifically for depression, there have been good signs of progress to apply MLT for neuroimaging data (Kambeitz et al., 2017) and this should be applied for future neurofeedback interventions.

## Conclusion

Despite the described limitations, the results of the present study suggest that bio- and neurofeedback constitutes a promising technique for the reduction of depressive symptomatology in many diverse populations, including patients with MDD. Given the technological advancements in biosensors, a great improvement of this kind of technique may be expected in the near future. Furthermore, these interventions could be consistently integrated into psychotherapeutic contexts (Lehrer, 2018), constituting together a potential alternative to the state-of-the-art developments in the treatment of depression (Hollon, Cohen, Singla, & Andrews, 2019).

The authors declare that the research was conducted in the absence of any commercial or financial relationships that could be construed as a potential conflict of interest.

## Supplementary material

For supplementary material accompanying this paper visit https://doi.org/10.1017/S0033291721004396. click here to view supplementary material
